# Temporal expression of avian β defensin 10 and cathelicidins in the yolk sac tissue of broiler and layer embryos

**DOI:** 10.1016/j.psj.2022.102334

**Published:** 2022-11-12

**Authors:** M. Jia, J.E. Fulton, E.A. Wong

**Affiliations:** ⁎ Department of Animal and Poultry Sciences, Virginia Tech, Blacksburg, VA 24061, USA; † Hy-Line International, Dallas Center, IA 50063, USA

**Keywords:** yolk sac tissue, avian β defensin, cathelicidin, broiler, layer

## Abstract

The yolk sac is a multifunctional organ, which not only participates in nutrient absorption, but also plays an important role in immune function. The objective of this study was to compare the mRNA abundance of avian β-defensin 10 (**AvBD10**) and 3 cathelicidins (CATH1, CATH2, and CATH3) in the yolk sac tissue (**YST**) of commercial broilers and white egg and brown egg commercial layers. AvBD10 and CATH mRNA abundance was analyzed using two-way ANOVA and Tukey's test, with *P* < 0.05 being considered significant. AvBD10 and CATH mRNA showed similar temporal expression patterns in the YST of both broiler and layers, with an increase from embryonic day (E) 7 to E9 through E13 followed by a decrease to day of hatch. AvBD10 mRNA showed a breed × age interaction with greater expression in the YST of both layers compared to broilers at E9 and E11. CATH1 mRNA was greater in the YST of brown egg layers than broilers. CATH2 mRNA showed a breed × age interaction, with greater expression in the YST of brown egg layers than broilers at E11. CATH3 mRNA showed no difference in the YST between layers and broilers. Because broilers and brown egg layers are genetically related, these results show that selection for production parameters (broiler vs. layer) and not genetic relatedness (white egg layer vs. brown egg layer and broilers) is the basis for the differences in AvBD10, CATH1, and CATH2 mRNA in the YST of broilers and layers. The yolk-free body weights of broiler embryos were greater than that of both brown and white egg layers from E9 to 17. One possible explanation is that the reduced expression of AvBD10, CATH1 and CATH2 mRNA in the YST of broilers compared to layers at E9 and 11 may be due to faster embryonic growth at the expense of host defense peptide expression in broilers compared to layers.

## INTRODUCTION

The yolk sac tissue (**YST**) is an extra-embryonic membrane that surrounds the egg yolk and provides essential functions for the embryo, while its organs are developing (Wong and Uni, 2021). One of the key functions of the YST is immunity. The YST transports IgY in the yolk to the blood circulation of the embryo (Hincke et al., 2019) and also expresses high mRNA levels of the host defense peptide (**HDP**) avian β-defensin (**AvBD**) 10 (Zhang and Wong, 2019). Transfer of IgY and expression of a HDP in the YST would provide immunity for the newly hatched chick before its own immune system matures.

There are 14 AvBD genes in chickens, which are clustered on chromosome 3 (Cheng et al., 2015). All AvBD peptides show antimicrobial activity, with AvBD5, 8, 10, and 12 showing the greatest activity (Lee et al., 2016). In the YST of chicks, AvBD10 mRNA was the most abundantly expressed and was localized to endodermal epithelial cells by in situ hybridization (Zhang and Wong, 2019). AvBD10 mRNA in the YST increased from embryonic day (**E**) 7 to E9, remained elevated from E9 to E13 and then declined from E13 to E19.

The cathelicidins (**CATH**) are another major family of HDP that are expressed in mammals and avians and show antimicrobial activity against a wide spectrum of bacteria (Van Harten et al., 2018; Wang et al., 2020). In chickens, there are four cathelicidins: CATH 1, 2, 3, and B1, which are clustered on chromosome 2 (Xiao et al., 2006; Cuperus et al., 2013). CATH1 and CATH3 are the most closely related with greater than 90% sequence identity (Zhang and Sunkara, 2014). CATHB1 is expressed predominantly in the bursa. CATH1, CATH2 and CATH3 mRNA were expressed in whole embryos and showed similar expression patterns with increases from E3 to E6 and E9 (Meade et al., 2009). All CATH peptides show similar antimicrobial activity against pathogens (Xiao et al., 2006; Lee et al., 2016). Expression of CATH mRNA in the YST, however, has not been reported.

It is often assumed that differences in gene expression between broilers and layers are due to the different selection parameters for these chickens: rapid growth rate of broilers vs. high egg production rate of layers. However, this assumption is potentially confounded by breed differences as broilers and white egg layers were developed from very different breeds. Commercial broilers were originally developed from multiple heavy dual-purpose breeds (brown shell eggs), which included the White Plymouth Rock (Crawford, 1990). The genetic basis of commercial brown egg layers includes some breeds in common with broiler origins. In contrast, commercial white egg layers are produced solely by the White Leghorn breed. Genetic diversity studies confirmed the close relationship between broilers and brown egg layers and the greater genetic distance of White Leghorns (Malomane et al., 2019).

Van der Most et al. (2011) investigated a potential tradeoff between growth and immune function in poultry that had been divergently selected for growth. Their meta-analysis showed that selection for growth compromised immune function. The objective of this study was to compare the temporal expression patterns of CATH1, CATH2, CATH3, and AvBD10 mRNA in the YST of embryos from broilers and layers. This study utilized 3 distinct types of chickens, broilers, and both white and brown egg layers. Differences between broilers vs. layers would implicate the impact of different selection parameters, while differences between a white egg breed vs. a brown egg breed would imply that they are influenced primarily by breed differences.

## MATERIALS AND METHODS

### Egg Incubation and Tissue Collection

Seventy-five broiler eggs (Cobb 500) were obtained from a commercial hatchery in Harrisonburg, VA. Seventy-five eggs from brown egg layers (HYB, Rhode Island Red × White Plymouth Rock) and 75 eggs from white egg layers (W36, White Leghorn) were obtained from a commercial Hy-Line hatchery (Elizabethtown, PA). Eggs were randomly placed and incubated simultaneously in 3 OvaEasy 190 incubators set at 37.5°C (Brinsea Products Inc, Titusville, FL). At embryonic age E7, E9, E11, E13, E15, E17, and E19, 6 viable eggs from each breed were randomly selected. Embryos were euthanized by cervical dislocation and yolk-free body weights (**YFBW**) of embryos were measured. At DOH, 6 chicks from each breed were weighed and euthanized. Two small pieces of YST were collected from each embryo, rinsed with cold 1 × PBS, minced, and placed into cryotubes, snap frozen in liquid nitrogen, and stored at −80°C for relative gene expression analysis. All animal procedures were approved by the Virginia Tech Institutional Animal Care and Use Committee.

### RNA Isolation and Relative Gene Expression Analysis

Approximately 25 mg of YST were homogenized in TRI Reagent (Molecular Research Center, Inc. Cincinnati, OH) using a tissue lyser. Total RNA was isolated using the Direct-zol RNA MiniPrep kit (Zymo Research, Irvine, CA). The concentration and purity of the RNA samples were analyzed using a Nanodrop-1000 (Thermo Fisher Scientific, Waltham, MA). One μg of total RNA was used to synthesize cDNA using the High-Capacity cDNA Reverse Transcription Kit (Thermo Fisher Scientific). cDNA was diluted 1:20 and used for real-time quantitative PCR (**qPCR**). Each sample was run in duplicate. Each well consisted of 5 μL SYBR green master mix (Thermo Fisher Scientific), 1 μL forward and reverse primers (5 μM), and 1.5 μL diluted cDNA and DEPC water. qPCR was performed with an Applied Biosystems 7500 Fast Real-time PCR system (Thermo Fisher Scientific) using the default fast program: 95°C for 20 s, 40 cycles of 90°C for 3 s and 60°C for 30 s. Primers for qPCR for AvBD10, CATH1, CATH2, CATH3, and CATHB1 are listed in Table 1. The stability of mRNA expression of 5 reference genes for all YST samples from all 3 types of chicks was tested using RefFinder (Xie et al., 2012). The five reference genes included 2 ribosomal proteins (RPLP0 and RPLP1), glyceraldehyde-3-phosphate dehydrogenase (**GAPDH**), β-actin (**ACTB**), and TATA-box binding protein associated factor 4 (**TAF4**). Only RPLP0, ACTB, and TAF4 were selected to be used for relative gene expression analysis due to their greater expression stability. The geometric mean of the Ct values of RPLP0, ACTB, and TAF4 was used to calculate ΔCt. The average ΔCt for broilers at E7 was used as the calibrator. Relative gene expression was determined by the $2^{-\Delta \Delta Ct}$ method (Schmittgen and Livak, 2008).

**Table 1:** Primers for real time quantitative PCR.

Gene^a^	Forward primer (5′→3′)	Reverse primer (5′→3′)	Amplicon size (bp)	Gene Accession number
AvBD10^2^	CAGACCCACTTTTCCCTGACA	CCCAGCACGGCAGAAATT	64	NM_001001609.2
CATH1^1^	TGGCCGCTGGTCATCAG	TTCTTGATCGCCCGGTAGAG	59	NM_001001605.3
CATH2^1^	CCGGGCGTCGATCTGA	GGTGCACTCTGTCTCCATGATG	63	NM_001024830.2
CATH3^1^	CGATGTCACCTGCGTGGAC	TTCTCCTGATGGCTTTGTAGAGGT	122	NM_001311177.1
CATHB1^1^	GCAGCTGTGCGGTTGCT	CAGCCGTAGGATGCATGGA	54	NM_001271172.1
RPLP0^2^	GCGATTGCTCCCTGTGATG	TCTCAGGTCCGAGACCAGTGT	59	NM_204987.2
RPLP1^2^	TCTCCACGACGACGAAGTCA	CCGCCGCCTTGATGAG	62	NM_205322.1
GAPDH^3^	GCCGTCCTCTCTGGCAAAG	TGTAAACCATGTAGTTCAGATCGATGA	73	NM_204305.1
ACTB^3^	GTCCACCGCAAATGCTTCTAA	TGCGCATTTATGGGTTTTGTT	78	NM_205518.1
TAF4^4^	CCAACTTGACTGCATTAGCTGC	TCGCGTAAACTGTCTGGTTGT	146	XM_417400.6

a Abbreviations: ACTB, β-actin; AvBD10, avian β-defensin 10; CATH, cathelicidin; GAPDH, glyceraldehyde-3-phosphate dehydrogenase; RPLP0, Ribosomal protein lateral stalk subunit P0; RPLP1, Ribosomal protein lateral stalk subunit P1; TAF4, TATA-box binding protein associated factor 4. Primers were designed using

1 Primer express 3.0 or were from

2 Zhang and Wong (2019),

3 Liu et al. (2020) and

4 Mazanko et al. (2019).

### Statistical Analysis

Differences in weight of yolk-free embryos or day of hatch chicks between breeds for each embryonic day were analyzed by one-way ANOVA and Tukey's test. Differences in relative gene expression between breeds and ages were analyzed by two-way ANOVA and Tukey's test using JMP Pro 15 (SAS Institute Inc., Cary, NC).

## RESULTS

### Body Weight of Layer and Broiler Embryos

**Figure 1:**
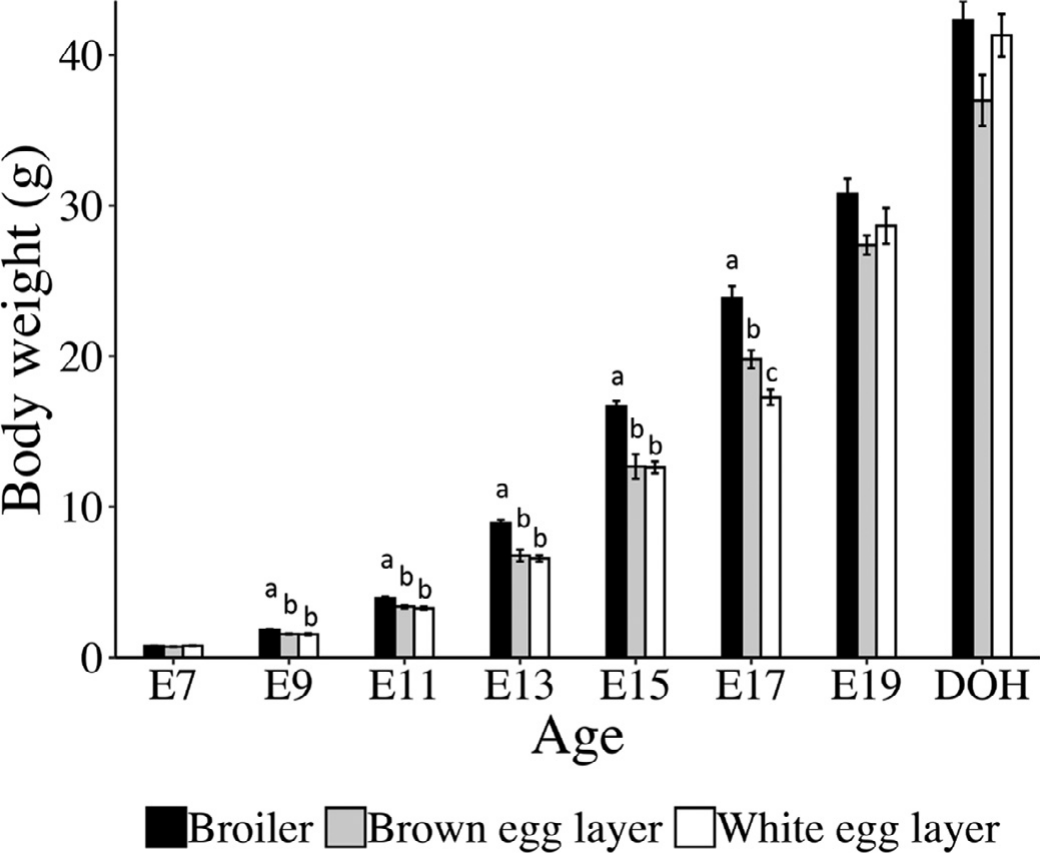
Body weights of embryos and chicks at day of hatch for broilers, brown egg layers and white egg layers. Yolk free body weights at embryonic day (E) 7 to E19 and body weight of chicks at day of hatch (DOH) were recorded. Bars show mean ± individual SE (n = 6). Statistical analysis used one-way ANOVA and Tukey's test. Different letters (a-c) indicate differences between breeds within an age (*P* < 0.05).

The growth of broiler and layer chicks was assessed by measuring YFBW of embryos from E7 to E19 and BW of chicks at DOH (Figure 1). Egg weights at the start of incubation were: broiler eggs (60.7 ± 0.5 g), brown eggs layer (58.6 ±0.5 g), and white eggs layer (63.8 ±0.5 g). One-way ANOVA showed that the weights of white eggs layer > broiler eggs > brown eggs layer, consistent with the pre-incubation egg weight distribution. At E7, there was no difference in YFBW between broiler and layer embryos. At E9, E11, E13, E15, and E17, the YFBW of broiler embryos were greater than both layer embryos. The YFBW of layers were the same except for at E17 when brown egg layers had greater YFBW than white egg layers. By E19 and DOH the YFBW and BW, respectively, of the layers and broilers were not different, likely because egg size restricted further growth. Consistent with this idea, brown eggs had the lowest egg weights and numerically lower BW at E19 and DOH.

### AvBD10 and CATH mRNA Expression in the Yolk Sac Tissue of Broilers and Layers

**Figure 2:**
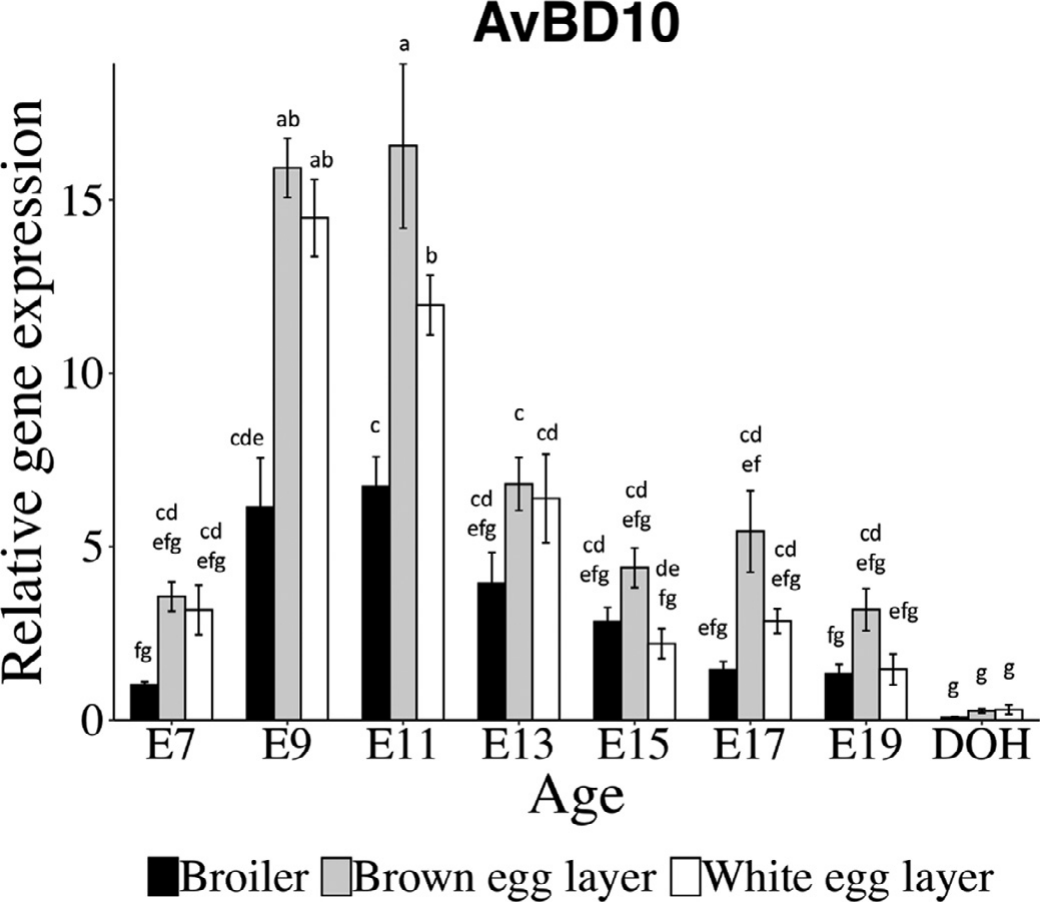
Relative gene expression for avian β-defensin 10 (AvBD10) in the yolk sac tissue of broilers and layers. Bars show mean ± individual SE for each gene (n = 6). Statistical analysis used two-way ANOVA and Tukey's test. Different letters (a-g) indicate differences between breeds or ages (*P* < 0.05).

Expression of AvBD10, CATH1, CATH2, and CATH3 mRNA were analyzed by two-way ANOVA for age, breed, and breed × age interaction (Table 2). When there was an interaction (AvBD10 and CATH2), the results were also displayed as a graph. AvBD10 mRNA in the YST of broilers, brown egg and white egg layers all showed similar temporal patterns with low levels at E7, a peak at E9/11 and a low level at DOH (Figure 2). There was a breed × age interaction (*P* < 0.001) for AvBD10 mRNA with all 3 chick types showing different levels of expression (Table 2). For broilers, AvBD10 mRNA increased from E7 to E9 and E11, decreased from E11 to E17 and was unchanged from E17 to DOH. By contrast, AvBD10 mRNA for both brown egg and white egg layers increased from E7 to E9 and E11, decreased from E11 to E13 and from E13 to DOH. At E9, AvBD10 mRNA in the YST was not different between the 2 layers; whereas at E11, AvBD10 mRNA was greater in brown egg layers than white egg layers (Figure 1). Overall, AvBD10 mRNA in the YST was greater in both layers compared to broilers (Table 2).

**Table 2:** Expression of avian β-defensin 10 (AvBD10) and cathelicidin (CATH 1-3) mRNA in the yolk sac tissue of broilers and layers from embryonic day 7 (E7) to day of hatch (DOH).

	Genes^1^			
	AvBD10	CATH1	CATH2	CATH3
Breed^2^				
Broiler	2.9^c^	5.5^b^	5.4^b^	5.5
Brown egg layer	7.2^a^	7.7^a^	7.9^a^	6.5
White egg layer	5.4^b^	7.0^ab^	7.7^a^	5.4
SEM	0.3	0.56	0.58	0.46
*P*-value	<0.0001	0.01	0.004	0.17
Age^3^				
E7	2.6^c^	1.6^c^	2.5^ef^	1.6^d^
E9	12.7^a^	6.0^b^	7.9^cd^	6.3b^c^
E11	11.8^a^	10.6^a^	14.3^a^	9.8^a^
E13	5.7^b^	10.5^a^	12.1^ab^	8.7^ab^
E15	3.1^c^	7.3^ab^	9.3^bc^	6.6^abc^
E17	3.2^c^	10.3^a^	6.1^cde^	8.2^abc^
E19	2.0^cd^	7.5^ab^	3.9^def^	5.1^c^
DOH	0.2^d^	0.4^c^	0.2^f^	0.3^d^
SEM	0.49	0.9	0.94	0.76
*P*-value	<0.0001	<0.0001	<0.0001	<0.0001
B × A Interaction				
*P*-value	<0.0001	0.29	0.002	0.41

1 Statistical analysis used two-way ANOVA and Tukey's test. Different letters (a-f)indicate differences (*P* < 0.05) between breeds (B) or ages (A).

2 Main effects of breed: average of values of all ages for each breed.

3 Main effects of age: average of values of all breeds for each age. Chick ages included embryonic days 7 (E7), 9 (E9), 11 (E11), 13 (E13), 15 (E15), 17, (E17), 19 (E19) and day of hatch (DOH).

For CATH1 mRNA, there was no breed × age interaction, but there were main effects of age and breed (Table 2). CATH1 mRNA showed a similar pattern of change across all 3 types of chicks, starting at a low level at E7 that increased to E11, no change from E11 to E19, and then a decrease from E19 to DOH. CATH1 mRNA was greater in the YST of brown egg layers compared to broilers, with white egg layers intermediate.

**Figure 3:**
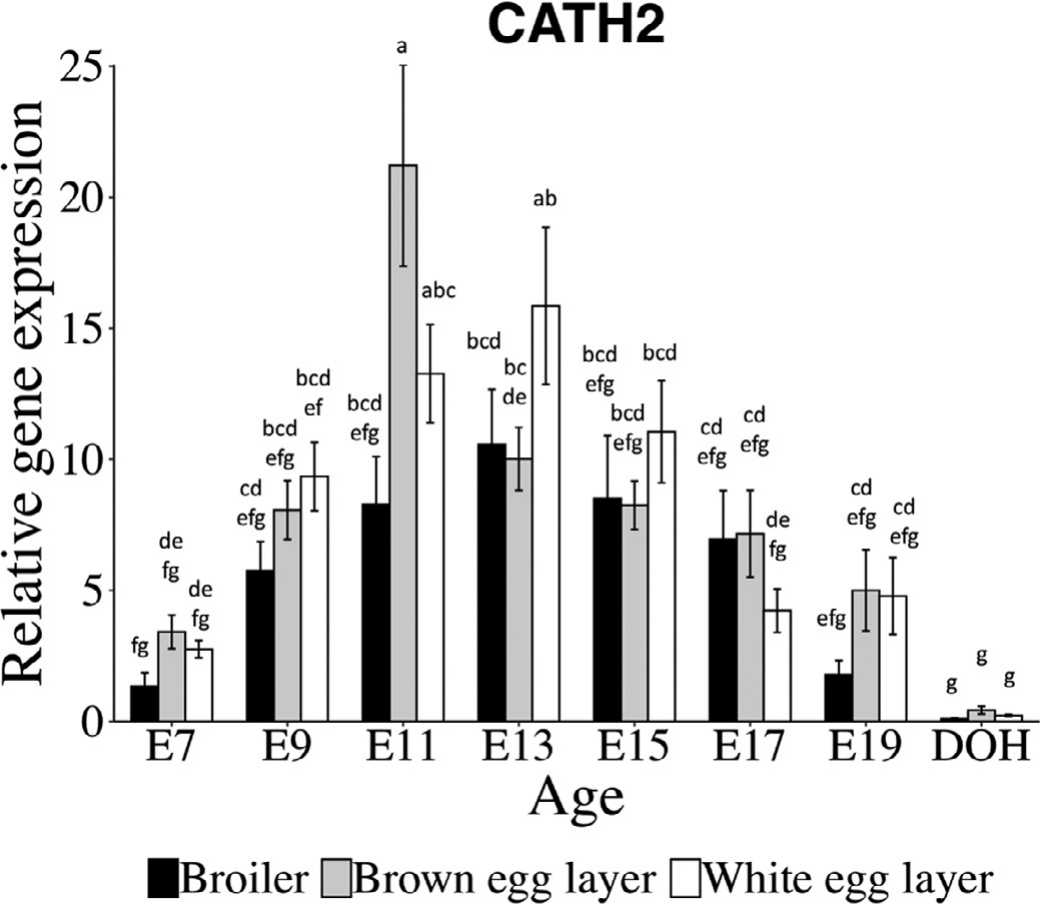
Relative gene expression for cathelicidin 2 (CATH2) in the yolk sac tissue of broilers and layers. Bars show mean ± individual SE for each gene (n = 6). Statistical analysis used two-way ANOVA and Tukey's test. Different letters (a-g) indicate differences between breeds or ages (*P* < 0.05).

CATH2 mRNA showed a similar temporal pattern of an increase from E7 to E11 and a decrease from E11 to DOH (Table 2). There was a breed × age interaction (*P* = 0.002) for CATH2 mRNA (Figure 3). CATH2 mRNA in the YST of broilers increased from E7 to E13 and then decreased from E13 to E19 and DOH. By contrast, CATH2 mRNA in the YST of brown egg layers increased from E7 to E11, decreased from E11 to E13 and from E13 to DOH; while CATH2 mRNA for white egg layers increased from E7 to E11 and E13 and then decreased from E13 to E17 and from E17 to DOH. At E11, CATH2 mRNA was greater in the YST of brown egg layers than broilers, with white egg layers intermediate. There was no difference for CATH2 mRNA between broilers and layers at any other time points. Overall, CATH2 mRNA was greater in the YST of both layers compared to broilers (Table 2).

For CATH3 mRNA there was no breed × age interaction, but there was a main effect of age (Table 2). CATH3 mRNA similarly showed the same temporal expression pattern with an increase from E7 to E9 and E11, no change from E11 to E17, and a decrease from E17 to DOH.

CATHB1 mRNA was not detected in the YST of either broiler or layer embryos at any age (data not shown).

## DISCUSSION

The egg contents must provide not only nutrition but also protection from pathogens for the developing embryo. The egg white contains ovotransferrin and lysozyme, both of which have antimicrobial properties; while the yolk contains hen-derived antibodies (Wong and Uni, 2021). Zhang and Wong (2019) had previously shown that AvBD10 mRNA was expressed in the YST. The current study confirmed AvBD10 mRNA expression in the YST and additionally showed that CATH1, CATH2, and CATH3 mRNA were also expressed in the YST. The combined antimicrobial action of biomolecules in the egg white, yolk, and YST creates a protective environment for the developing embryo.

The profiles of AvBD10 and CATH mRNA all showed peak expression during mid embryogenesis (E9 to E13) in the YST of both broilers and layers. The decline in AvBD10 and CATH mRNA during late incubation would be consistent with the beginning of degradation of the YST prior to internalization. Peak expression during mid incubation would provide protection for the developing embryo from any pathogens that may be present in the egg yolk while the immune organs of the embryo are in the process of developing and maturing. It is unknown what the trigger might be for this increase in AvBD10 and CATH mRNA, but it is unlikely due to contamination in the eggs because this induction occurred consistently with a previous trial using broilers (Zhang and Wong, 2019) and the current trial using both broiler and brown and white egg layers from different commercial hatcheries. This is most likely a genetically programmed event. Meade et al. (2009) reported the temporal expression patterns for AvBDs and CATHs in whole Cobb embryos at E3, E6, and E9. AvBD5, AvBD9, and AvBD10 mRNA increased 25-, 164- and 87-fold, respectively, from E3 to E9 in whole embryos while CATH1, CATH2, and CATH3 mRNA increased 7-, 6-, and 9-fold, respectively, from E3 to E6 (Meade et al., 2009). Thus, in the developing embryo, immune protection is from a combination of AvBD and CATH expression by the embryo during early incubation (E3 to E9) and by the YST during mid incubation (E9 to E13).

Genetic distance analysis showed that brown layers are genetically more closely related to commercial broilers than to commercial White Leghorns (Malomane et al., 2019). This is consistent with the known origin of commercial brown egg layers, being derived from some of the same breeds used to develop the commercial broiler (Crawford, 1990). The brown and white egg layers both had AvBD10, CATH1, and CATH2 mRNA levels greater than broilers, and were more similar to each other than to broilers. This suggests that the differences observed in this study were not due to genetic differences per se (brown egg layers and broiler breeds vs. White Leghorn breed), but were more likely due to the selection for specific performance traits, that is, rapid early growth rate for broilers vs. high egg numbers and extended egg production for layers.

The difference in HDP mRNA expression profiles in the YST between broilers and layers may be due to selection for rapid growth. In this study, broiler embryos had greater YFBW than layer embryos between E9 and E17, which was also reported by Ohta et al. (2004). AvBD10, CATH1, and CATH2 mRNA, however, were lower in the YST of broiler embryos compared with layer embryos during this period. One possible explanation is that the increased early growth rate of broilers may come at a cost of decreased expression of HDPs in the YST. Because there is a finite amount of nutrients in an egg, energy may be directed towards growth instead of host defense for broiler embryos. This hypothesis, however, needs further investigation.

There was elevated expression of CATH1 and CATH2 mRNA in the YST of layers compared to broilers; whereas CATH3 mRNA showed no difference. Expression of CATHB1 mRNA was not found in the YST (data not shown), confirming that it is predominantly expressed in the bursa (Achanta et al., 2012). The CATH genes are organized as a cluster on chromosome 2 in the order of CATH1-CATHB1-CATH2-CATH3, with CATH3 transcribed from the opposite strand as the other CATH genes (Cuperus et al., 2013; Cheng et al., 2015). CATH1 and CATH 3 showed the greatest sequence identity (>90%) and are thought to have arisen by gene duplication (Xiao et al., 2006; Zhang and Sunkara, 2014). One possible explanation for the difference between CATH1/CATH2 and CATH3 mRNA expression in the YST of broilers and layers may be because CATH1 and CATH3 have similar amino acid sequences and induced expression of both would be redundant. A second possibility is that there may have been a change in the regulatory elements of the CATH1 and CATH3 genes during gene duplication.

In conclusion, the temporal expression patterns for AvBD10 and CATH1, CATH2, and CATH3 mRNA were similar in the YST of broiler and layer embryos, with an increase from E7 to E9/E11, followed by a decline to DOH. The peak HDP expression during mid embryogenesis would protect the embryo from pathogens present in the egg, while the immune organs of the embryo are in the process of maturing. However, layers, especially brown egg layers, had greater AvBD10, CATH1, and CATH2 mRNA in the YST compared to broilers. The increased early expression of AVBD10, CATH1, and CATH2 mRNA in the YST of layer compared to broiler embryos was inversely related to the lower YFBW of layer compared to broiler embryos. This suggests that during mid embryogenesis, energy may be directed towards growth at the expense of host defense for broilers. These results further indicate that differences between expression levels of HDPs in the YST of layer and broiler embryos are more likely due to the different selection parameters of growth (broilers) vs. egg production (layers) rather than breed differences (broilers and brown egg layers vs. White Leghorn). The unique design using broilers and brown and white egg layers would allow future studies to also dissect differences due to genetics or selection.
